# Prospective blinded surveillance screening of Swedish women with increased hereditary risk of breast cancer

**DOI:** 10.1007/s10549-017-4639-0

**Published:** 2018-01-09

**Authors:** Annelie Liljegren, Anna von Wachenfeldt, Edward Azavedo, Sandra Eloranta, Helene Grundström, Anne Kinhult Ståhlbom, Ann Sundbom, Per Sundén, Gunilla Svane, Dieter Ulitzsch, Brita Arver

**Affiliations:** 1 Department of Oncology-Pathology, Karolinska University Hospital, Karolinska Institutet, Radiumhemmet, 171 76 Stockholm, Sweden; 2 Department of Oncology-Pathology, Karolinska University Hospital/Södersjukhuset, Stockholm, Sweden; 3 Department of Radiology, Karolinska University Hospital, Karolinska Institutet, Stockholm, Sweden; 4 Scandinavian Development Services, Danderyd, Sweden; 5 Department of Radiology, Danderyd Hospital, Stockholm, Sweden; 6 Department of Radiology, Södersjukhuset, Stockholm, Sweden

**Keywords:** Hereditary breast cancer, Surveillance, Prevention program, Mammography, Ultrasound, BRCA1, BRCA2

## Abstract

**Purpose:**

To evaluate the sensitivity and specificity of different screening modalities in women with a family history of breast cancer.

**Methods:**

Our blinded, prospective, comparative cohort analysis included three types of screening, mammography, ultrasound, and clinical breast examination once per year for 6 years. Eligible patients for this study were healthy women with ≥ 17% lifetime risk of breast cancer or with a mutation in *BRCA1* or *BRCA2*.

**Results:**

A total of 632 women were screened between 2002 and 2012 (each for 6 years). During the study, 30 women were diagnosed with breast cancer, with 10 of these diagnoses occurring between screening visits, and six of the 10 diagnosed women were gene carriers. The clinical presentation for the women diagnosed with breast cancer was followed until 2017. No consistent patterns for the diagnostic capacity of the different screening modalities were found, although mammography showed low sensitivity, whereas ultrasound showed better sensitivity in three of the six rounds. The specificity was high in mammography and improved in ultrasound over time. Most importantly, clinical breast examination provided no additional information toward the diagnosis of breast cancer.

**Conclusion:**

Neither mammography nor ultrasound performed yearly were sensitive enough as standalone modalities, although high specificity was confirmed. Our findings indicate that high risk (> 29% life time risk) individuals and gene carriers can be screened biannually, using the same protocol as used in mutation carriers. Our results also suggest that low-risk groups (< 20%) may continue to be referred to population mammography screening program, while clinical breast examination may be omitted in all risk groups, and could be optional in gene carriers.

**Electronic supplementary material:**

The online version of this article (10.1007/s10549-017-4639-0) contains supplementary material, which is available to authorized users.

## Introduction

Worldwide awareness of risk factors associated with a family history of breast cancer is increasing. Women at increased risk (often defined as doubled lifetime risk) are recommended to obtain regular surveillance after genetic counseling and risk assessment [7, 15]. Women with mutations in the *BRCA1*, *BRCA2*, *PALB2*, or *TP53* genes who are at considerably high risk for breast cancer are also informed about the possibility of risk-reducing surgery. Surveillance includes mammography, which may be used in combination with breast ultrasound, breast magnetic resonance imaging, and clinical breast examination. Breast self-examination is also advocated. Limited sensitivity in detecting breast cancer with the use of only mammography (32–41%) [10–13, 20, 21] or only ultrasound (32–60%) [16, 19] has been reported. Mammography used in conjunction with ultrasound has a higher, sensitivity ranging of 49% [11] to 77.5% [5] than mammography or ultrasound alone. The sensitivity of magnetic resonance imaging is superior to that of the other imaging methods [10–13] especially in young women and in *BRCA1*- *or BRCA2*-mutation carriers [20]. International guidelines advocate annual mammography to women with moderate (17–29%) life time risk and annual mammography and magnetic resonance imaging to *BRCA1*- or *BRCA2* mutation carriers [15]. However, there has been no blinded study following a large cohort of women over multiple years to assess the importance of these test modalities in combination. The majority of the published surveillance studies report a follow-up of 1–4 years [15] and there is a lack of reports of long time surveillance in a cohort of women at increased risk due to hereditary breast cancer.

Therefore, we designed this blinded, prospective study with the aim to evaluate the sensitivity and specificity, i.e., the diagnostic capacity/effectiveness of the different screening tests upon imaging, of the different screening modalities (mammography, ultrasound, clinical breast examination) in women with a family history of breast cancer. The study compared screenings for 632 women using three types of screening, mammography, ultrasound, and clinical breast examination once per year for 6 years and ran from 2001 to 2012. Eligible patients for this study were healthy women with ≥ 17% lifetime risk of breast cancer or with a mutation in *BRCA1* or *BRCA2.*

## Materials and methods

### Study design and study center

This prospective, comparative cohort study aimed to compare the diagnostic capacity of screening modalities among women with an increased risk of breast cancer due to family history. The surveillance included three types of screening, mammography, ultrasound, and clinical breast examination once per year for 6 years for each participant. Enrollment of the women in the study took place between 2002 and 2006 with the final screening visit for the last included patient in 2012. For patients that were diagnosed with breast cancer within the study, follow-up data regarding recurrences and death were collected from medical records up to October 2017 in order to give a descriptive view of survival so far. As only one magnetic resonance imaging unit in a developing phase was present in our region when this study was conducted, magnetic resonance imaging screening was not included in our study although since 2006, magnetic resonance imaging has been used for *BRCA1*/*2* mutation carriers and in individuals with a medical history of breast cancer according to international guidelines. Only descriptive data about the magnetic resonance imaging findings are presented in this report. Study participants were recruited from the Familial Cancer Centre, Oncology Department, Karolinska University Hospital. This centre has three site locations in Stockholm: Danderyds Hospital (Site 1), Karolinska University Hospital (Site 2), and Södersjukhuset (Site 3). Written informed consent was obtained from all participants, and the Ethics Committee at Karolinska Institutet approved the study on 15-10-2001 (no. 01-065).

### Patients

Inclusion criteria: healthy women between the ages of 25 and 60 underwent genetic counseling and risk assessment. Women with ≥ 17% lifetime risk of breast cancer according to Claus tables [6], or women with a family history indicating an autosomal dominant disease of breast cancer, were eligible for the study. Those with a personal history of breast or ovarian cancer and a 5-year disease-free interval in combination with a family history for breast cancer were also included. In addition, healthy women with a mutation in *BRCA1*, *BRCA2*, *PTEN*, or *TP53* were eligible. A normal mammogram 1 year before the first screening round was mandatory.

Exclusion criteria: Women with no known mutation in the family were not included if they were > 10 years younger than the earliest age in which a family member was affected with breast cancer. Mutation carriers were not eligible if younger than 25 years of age.

A total of 656 women with a normal mammogram were initially included in the study. Twenty-four women declined participation before entering screening rounds.

The final number of women enrolled who started screening round 1 was 632 with a mean age of 44.1 (range 25–60). Over the course of 6 years, 95 women discontinued the study due to prophylactic mastectomy (*n* = 46), logistics (*n* = 13), migration (*n* = 12), tested as a non-carrier (*n* = 7), non-cancer-related death (*n* = 3), and lost contact (*n* = 12). Of the 3792 planned screening visits, a total of 3478 screenings were performed (92%).

### Genetic investigation

The counseling procedure included a pedigree of family history with medical records confirming cancer diagnoses and death certificates or data from the Swedish Cancer Registry. Genetic screening of the *BRCA1/2* genes was offered to families having one of the following characteristics according to the national guidelines: (1) at least three cases of breast or ovarian cancer in first- or second-degree relatives (with at least one relative younger than 50 at diagnosis); (2) two cases of breast or ovarian cancer in first- or second-degree relatives (at least one relative below age 40 at diagnosis); (3) one individual with breast or ovarian cancer diagnosed when less than 35 years of age; (4) close relatives with the combination of breast and ovarian cancer, regardless of the age of onset.

### Risk assessment

In this study, the Claus risk tables [6] were used to determine lifetime risk of breast cancer in families with one or two close relatives with breast cancer. All pedigrees were grouped in relation to hereditary patterns (supplementary Table 1). The pedigrees were classified according to each study participant’s life time risk of breast cancer, and four groups were defined: risk group 1—moderate risk of breast cancer; risk group 2—high risk of breast cancer; risk group 3—medical history of breast or ovarian cancer and at least one close relative with breast or ovarian cancer; risk group 4—very high risk, mutation carriers with or without a previous medical history of breast or ovarian cancer.

**Table 1:** Baseline data of the study group

	The whole study population *n* = 632		Risk group 1 *n* = 176		Risk group 2 *n* = 384		Risk group 3 *n* = 26		Risk group 4 *n* = 46	
Mean age (SD)	44.1 (8.4)		43.1 (8.0)		44.3 (8.4)		51.6 (6.6)		41.8 (8.9)	
	No.	%	No.	%	No.	%	No.	%	No.	%
Social status										
Married or living with partner	471	77	136	80	288	76	13	54	34	77
Single with children	62	10	13	8	39	10	5	21	5	11
Single	77	13	19	11	47	12	6	25	5	11
Education										
Junior high school	30	5	5	3	22	6	2	8	1	2
High school	105	17	33	20	57	15	5	21	10	23
Blue collar	94	15	19	11	61	16	3	13	11	25
Academic degree	383	63	110	66	237	63	14	58	22	50
Health related factors										
History of smoking	362	59	94	56	230	61	13	52	25	57
Number of years of smoking (mean) (SD)	7.5 (10.0)		7.2 (10.0)		7.7 (10.0)		5.6 (8.8)		8.0 (10.0)	
Number of women drinking alcohol once/week or more	514	84	139	84	316	83	21	84	38	84
Exercise minutes/week mean (SD)	306.3 (237.4)		303.5 (261.9)		307.7 (230.7)		279.2 (254.3)		320.8 (184.0)	
Reproductive history										
No history of pregnancy	89	15	24	15	55	15	2	8	8	18
1 pregnancy	67	11	12	7	45	12	3	12	7	16
2 or more pregnancies	449	74	129	78	272	73	20	80	29	66
Number of children (mean) (SD)	1.67 (1.7)		1.7 (1.2)		1.7 (1.1)		1.9 (1.1)		1.5 (1.4)	
Hormonal treatment to get pregnant	43	7	9	5	27	7	1	2	6	13
Breast feeding Yes	484	88	133	90	293	87	23	96	33	83
History of birth control pills Yes	538	88	153	91	327	87	20	83	38	86
Premenopausal^a^	489	77	151	86	299	78	8	31	31	67
Postmenopausal	143	23	25	14	85	22	18	69	15	33
Oophorectomy	54	9	10	6	20	5	7	28	17	40
Self-examination										
Self-examination: 1/month	300	49	78	47	185	50	12	50	25	57
Self-examination: 6 times/year	115	19	33	20	68	18	7	29	7	16
Self-examination: 4 times/year	135	22	37	22	84	23	4	17	10	23
Self-examination: never	57	9	19	11	35	9	1	4	2	5
Other risk related factors										
History of breast reduction operation	29	5	10	6	13	4	5	20	1	2

*SD* standard deviation

^a^Women with < 6 months since last menstruation *or* using hormone replacement therapy after oophorectomy *or* having progesteron uterine device were considered being premenopausal

The risk estimation model Breast and Ovarian Analysis of Disease Incidence and Carrier Estimation Algorithm [BOADICEA] has replaced the Claus tables in clinical practice during the last decade. Thus, the lifetime risk for all women in the study cohort was calculated retrospectively according to BOADICEA version 2 [3] to investigate if that selection of participants by Claus tables was comparable to that by BOADICEA.

### Breast assessment

The mammography, ultrasound, and clinical breast examination findings were scored on a five-point scale: 1 = normal, 2 = benign, 3 = possibly malignant, 4 = most probably malignant, and 5 = malignant. This is a modified version of mammographic findings described by Azavedo et al. and frequently used in Sweden [4]. Mammographic density was estimated according to Wolfe’s division of breast density into four groups of increasing density: N1, P1, P2, and DY [22]. When available, magnetic resonance images were categorized using the American College of Radiology Breast Imaging-Reporting and Data System (BI-RADS) categories and scored on a 5-point scale [1].

### Demographics and lifestyle assessment

Self-administered questionnaires were collected at the start of the study to record demographic characteristics, history of gynecological and/or breast surgery, reproductive history, hormonal treatment, menopausal status, body mass index, smoking and alcohol habits, physical activity, and habits of self-examination.

### Procedure

Mammography and ultrasound were performed no < 4 weeks apart and before the clinical breast examination that was done within 4 weeks from the imaging. The most experienced ultrasound radiologists worked at Site 1 and all ultrasound procedures were performed here. Mammography was done either at Site 2 or 3. Clinical breast examination was performed at all three sites. To ensure the procedure was blinded, no communication among the radiologists and the clinicians involved in the study was allowed before the annual clinical breast examination. Additionally, no communication regarding findings from the screening was permitted between the radiologists and the study subject. After the clinical breast examination was performed, the results from the imaging modalities were disclosed and made available to all physicians involved in the diagnostic process. If there was an abnormality (i.e., code 3 or higher on any imaging examination modality), the study subject was referred for further diagnostics. Specifically, women with palpable lesions code 3 or more were referred for fine-needle biopsy. Referrals for cytology were optional for code 2 (code 2 = benign) but was not included in the statistics on the sensitivity and specificity analyses. In pregnant or lactating women, only ultrasound imaging was used.

**Fig. 1:**
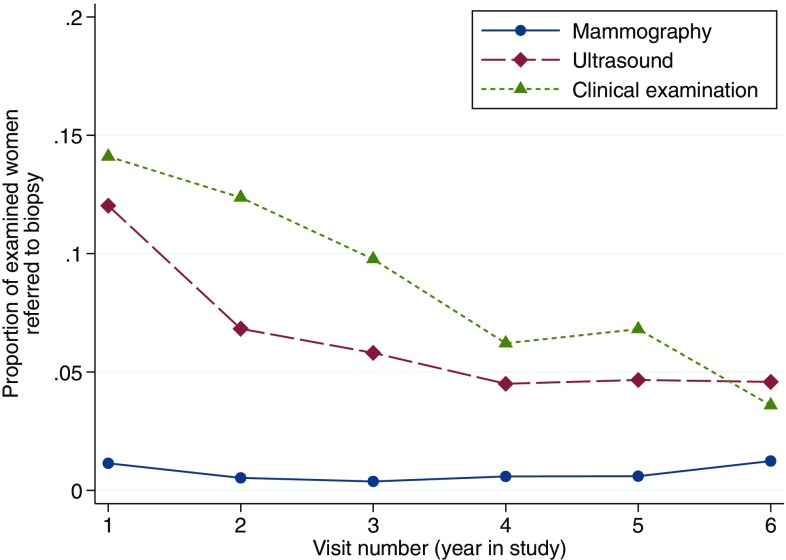
Proportion of examined women referred to biopsy at each screening round

During the 6-year screening program, 24 women (0.75% of all mammography imaging) were referred to further diagnostic investigation by the mammography breast radiologist, 213 (6.4% of all ultrasound imaging) by the ultrasound breast radiologist, and 302 (9.1% of all clinical breast examination) by the clinicians. Over time, there were fewer biopsy referrals from the clinicians and the ultrasound radiologists (Fig. 1).

### Screening modalities

A total of five experienced breast radiologists examined mammography images. Each mammogram was examined by two different breast radiologists. Mammography was performed with two views per breast, Medio-lateral-oblique and craniocaudal using an analogue technique at both sites. At Site 2, an Instrumentarium Diamond machine was used. At Site 3, the Siemens Mammomat 300 was used until 2006 when it was replaced by Siemens Mammomat 3000 Nova in January 2007.

Ultrasound was performed using Linear Array 8.7-MHz probes (L39, GE Logic 400). Since 2006, a high-resolution 5–13-MHz Linear Matrix Array probe (ML12, GE Logic 9) was increasingly used. Both breasts and the axillas were systematically examined by one of the three experienced ultrasound breast radiologists involved in this study.

For clinical breast examination, the breasts and regional lymphatic areas were examined with the study subject in both a sitting and a lying position. All clinical breast examinations were performed by one of the three oncologists at each of the three sites.

### Statistical analyses

At each screening round, the proportion of all women referred to biopsy after mammography, ultrasound, and clinical breast examination was determined. As a measurement of diagnostic capacity, the sensitivity and specificity were calculated at screening rounds 1 through 5. For each round, a 1-year detection time window was defined, which indicated the time until the next screening visit. Thus, for example, screening round 1 corresponds to the time period between the first and second screen set of mammography, ultrasound, and clinical breast examination; screening round 2 corresponds to the time between screen 2 and 3; and so forth. During this window, the number of suspected (i.e., referred to biopsy) and non-suspected (i.e., not referred to biopsy) breast cancers were compared to the number of true cases of breast cancers as confirmed by biopsy. The first detection window was from first screening at year 1 until second screening at year 2. The second detection window was from year 2 until third screening at year 3, etc. Women with suspected breast cancer could be independently referred to biopsy by way of the three diagnostic modalities. Cancers that were diagnosed between screening rounds and that were previously undetected by all three modalities were defined as “interval cancers.” For each screening round, the sensitivity was calculated as the number of women with a screening finding classified as code 3 or higher with a confirmed diagnosis of breast cancer divided by the total number of confirmed cases diagnosed within the detection window. The specificity was calculated as the number of breast cancer-free women who were not referred to biopsy at screening divided by the total number of women who were breast cancer-free (i.e., including false positives) during the same detection window.

The sensitivity and specificity at each screening round were used to produce non-parametric receiver operating characteristics curves for the three diagnostic modalities. The diagnostic capacity of each modality at each round was subsequently contrasted by calculating areas under the curve (AUC), including 95% confidence intervals. All descriptive statistics were performed with the SPSS for Windows program, version 16.0. Statistical analyses of sensitivity and specificity were performed using the roctab and roccomp functions in Stata 13 [18].

## Results

The baseline demographics of the women in the different risk groups and the results from the self-reported questionnaire administered at inclusion are presented in Table 1. The mean age was statistically significantly higher in risk group 3 (51.6) than in the other risk groups (41.8–43.1), *p* < 0.001.

### Cancer detection

In our study of at-risk women for breast cancer, thirty of the enrolled 632 women (4.7%) were diagnosed with breast cancer at some time during the 6-year screening protocol. Ten of these tumors occurred between screening visits. Among these ten cancers, two were found incidentally upon the histological examination following prophylactic mastectomy, and two cases of breast cancer were found in two women, who by mistake participated in the concurrent mammography population screening program. Among the 10 women with interval breast cancers, three women were known gene carriers before entering the study while three were identified as carriers after breast cancer diagnose. Twenty-five of the 30 women diagnosed with breast cancer had invasive tumors. Of these, nine (36%) were lymph node positive. Tumor characteristics, breast cancer recurrences, and years of survival up to October 2017 of the 30 patients diagnosed with breast cancer are presented in Table 2. Two patients died from breast cancer and two died from other cause during the follow-up time. One patient was diagnosed with contralateral breast cancer and one patient had a local recurrence. For further details see Table 2.

**Table 2:** Characteristics of all women and tumors with detected breast cancer

Patient ID	Screening visit	Age at BrC diagnose	Menopaus status	Risk group	BOADICEA life time risk^d^	Modalities that detected malignancy^c^	Density according to Wolfe	Type of surgery	Size (mm) DCIS Yes/no	Invasive histology type
Characteristics of the 20 women and tumors with a screened detected breast cancer										
1001	6	56	Post	History of BrC	na	MRI	P2	BCS	No	Ductal
1037	2	48	Post	History of BrC	na	US, CBE	P2	Extirpation of local recurrence	No	Ductal
1064	2	61	Post	History of BrC	na	US, CBE	DY	MAD	No	Ductal
1109	5	49	Pre	Moderate risk	20	XRM	P2	BCS	No	Ductal
1130	4	46	Post	Moderate risk	15	MG US, CBE	P1	MAD	No	Ductal
1167	1	48	Pre	Moderate risk	17	US	DY	MAD	Yes	Metaplastic
1183	4	40	Pre	*BRCA1*	76	US, MRI, CBE	P2	MAD	Yes	Unclassified low diff
2009	4	44	Pre	*BRCA1*	77	US (pregnant)	N1^b^	MAD	No	Ductal
2028	1	60	Post	High risk	46	US	P1	MAD	No	Ductal
2067	4	47	Pre	High risk^a^	19	US, CBE	P1	MAD	Yes	Ductal
2088	2	61	Post	History of BrC	na	MG, US	N1	BCS	No	Tubular
2132	5	56	Post	High risk	17	US	P2	BCS	No	Ductal
2151	5	54	Pre	Moderate risk	16	US	P1	BCS	No	Lobular
2239	4	55	Post	High risk^a^	28	MG, US	P2	MAD	No	Ductal
3023	4	58	Pre	*BRCA1*	60	US, CBE	P1	MAD	No	Ductal
3053	2	53	Post	High risk	28	MG	P2	MAD	30	na
3070	6	55	Post	High risk	29	MG	P2	BCS	2	na
3097	6	59	Post	High risk	23	US, CBE	P1	MAD	No	Ductal
3113	6	57	Post	High risk	21	US	P1	BCS	No	Lobular
3152	1	39	Pre	Moderate risk	17	MG, US, CBE	P2	MAD	43	Ductal

Patient ID	Invasive tumor size (mm)	Grade of invasive cancer	ER/PR	No. of positive lymph nodes	Proliferation (%)	HER2	TNM	Chemotherapy	Type of radiotherapy	Identified mutation after BrC-diagnose	Follow-up^g^
Characteristics of the 20 women and tumors with a screened detected breast cancer											
1001	8	2	pos/pos	0	25	neg	T1bN0M0	No	Breast		Relapse free 10 years
1037	16	2	pos/pos	0	10	neg	T1cN0M0	No	Tumor area		Relapse free 3 years, then metastases and dead after 10 years
1064	20	3	pos/neg	0	20	neg	T1cN0M0	No	None		Relapse free 13 years
1109	8	2	pos/pos	0	15	neg	T1bN0M0	No	Breast + boost		Relapse free 4 years, then death due to other cause
1130	15^e^	na	pos/pos	1	50	neg	T2N1M0	No	Breast + locoregional		Relapse free 9 years
1167	11	3	pos/pos	0	30	neg	T1cN0M0	Yes	None		Relapse free 11 years
1183	CR^f^	3	neg/neg	4	50	neg	T1cN1M0	No	Locoregional		Relapse free 1 year, then metastases and death after 2 years
2009	23	3	neg/neg	0	54	neg	T2N0M0	Yes	None		Relapse free 12 years
2028	9	2	pos/pos	0	< 2	neg	T1bN0M0	No	None		Contralateral BrC after 12 years but no signs of relapse 15 years after first BrC diagnose
2067	24	3	neg/neg	0	65	neg	T2N0M0	Yes	None	*BRCA1*	Relapse free 11 years
2088	10	1	pos/pos	0	3	neg	T1bN0M0	No	Breast		Relapse free 13 years
2132	9	2	pos/pos	0	10	neg	T1bN0M0	No	Breast		Relapse free 9 years
2151	20	2	pos/neg	0	2	neg	T1cN0M0	No	Breast		Relapse free 9 years
2239	17	3	pos/pos	1	23	neg	T1cN1M0	Yes	Locoregional	*BRCA1*	Relapse free 9 years
3023	9	3	neg/neg	0	60	neg	T1bN0M0	No	None		Relapse free 10 years, then death due to pancreas cancer 11 years after BrC diagnose
3053	no	na	na	na	na	na	DCIS	No	None		Relapse free 13 years
3070	no	na	na	na	na	na	DCIS	No	None		Relapse free 8 years
3097	20	2	pos/neg	1	20	neg	T1cN1M0	Yes	None		Relapse free 8 years
3113	10	2	pos/pos	0	18	neg	T1bN0M0	No	Breast		Relapse free 7 years
3152	22	3	pos/neg	2	23	neg	T2N1M0	Yes	None		Relapse free 11 years

Patient ID	Screening visit	Age at BrC diagnose	Menopaus status	Risk group	BOADICEA life time risk^d^	How the tumors were detected	Density previous year	Type of surgery	Size (mm) DCIS Yes/no	Invasive histology type
Characteristics of the 10 women and tumors detected in between screening rounds										
1051	na	46	na	Moderate risk^a^	19	MG—population screening	P1	Mastectomy	12	na
1178	na	41	na	Moderate risk	16	MG—population screening	na	MAD	No	Ductal
2020	na	37	Pre	*BRCA2*	88	Detected at PM	P2	PM	5	na
2181	na	39	Pre	Moderate risk	19	BSE	P2	MAD	No	Ductal
2208	na	60	Post	Moderate risk	21	BSE	P2	MAD	No	Lobular
2043	na	50	Pre	Moderate risk^a^	16	BSE	P2	MAD	No	Lobular
2252	na	49	Pre	Moderate risk	14	MR due to unspecific symptom	P2	BCS	40	Ductal
2269	na	30	Pre	*BRCA1*	93	BSE	P2	BCS	5	Ductal
3015	na	48	Pre	High risk^a^	31	BSE	P2	MAD	No	Ductal
3073	na	52	Pre	*BRCA2*	71	Minimal area with DCIS detected at PM	P2	PM	na	na

Patient ID	Invasive tumor size (mm)	Grade of invasive cancer	ER/PR	No. of positive lymph nodes	Proliferation (%)	HER2	TNM	Chemotherapy	Type of radiotherapy	Identified mutation after BrC-diagnose	Follow-up^g^
Characteristics of the 10 women and tumors detected in between screening rounds											
1051	no	na	na	na	na	na	DCIS	No	none	*BRCA2*	Relapse free 12 years
1178	9	3	neg/neg	3	90	neg	T1bN1M0	Yes	none		Relapse free 11 years
2020	no	na	na	na	na	na	DCIS	No	none		Relapse free 11 years
2181	80	2	pos/pos	0	80	neg	T3N0M0	Yes	Locoregional		Relapse free 12 years
2208	11	2	pos/neg	0	2	neg	T1cN0M0	No	None		Relapse free 10 years
2043	15	2	pos/pos	1	21	neg	T1cN1M0	Yes	None	*BRCA2*	Relapse free 14 years
2252	15	2	pos/pos	4	0	neg	T1cN1M0	Yes	Locoregional		Relapse free 8 years
2269	5	3	neg/neg	0	75	neg	T1aN0M0	Yes	Breast + boost		Relapse free 6 years
3015	20	3	pos/pos	3	1	pos	T1cN1M0	Yes	Locoregional	*BRCA1*	Relapse free 14 years
3073	no	na	na	na	na	na	DCIS	No	None		Relapse free 12 years, then local recurrence in left breast 12 mm ductal carcinoma

*MG* mammography, *CBE* clinical breast examination, *BSE* breast self-examination, *BrC* breast cancer, *MRI* magnetic resonance imaging, *MAD* mastectomy and axillary dissection, *PM* prophylactic mastectomy, *BCS* breast-conserving surgery, *pos* positive, *neg* negative, *TNM* tumor, node and metastasis staging for breast cancer

^a^Mutation was found after BrC

^b^Density previous screening round

^c^MRI was only performed in two patients; 1001, 1183

^d^Boadicea was calculated retrospectively

^e^35 mm before neoadjuvant chemotherapy

^f^17 mm before neoadjuvant chemotherapy, na; non-applicable

^g^Information from medical records October 2017

### Sensitivity, specificity, and area under the curve for the three screening modalities

In screening round 1 (the detection window between year 1 and year 2), the diagnostic capacity of mammography and ultrasound was similar with an AUC for mammography of 0.57 (95% CI 0.43–0.71) and for ultrasound of 0.58 (95% CI 0.40–0.76). However, the AUC for clinical breast examination (0.42, 95% CI 0.42–0.44) was significantly lower than that for mammography and ultrasound (*p* = 0.0045).

In screening round 2 (the detection window between year 2 and year 3), there was no difference in diagnostic capacity between the three screening modalities.

**Fig. 2:**
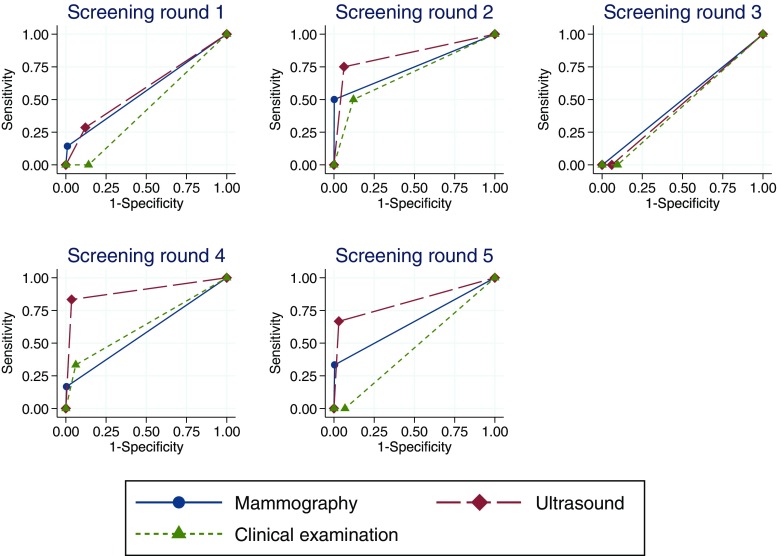
Sensitivity and specificity of mammography, ultrasound, and clinical breast examination at each screening round

In screening round 3, all three modalities resulted in AUCs of 0.50 or lower. Furthermore, the cancers that occurred during this time window were all interval cancers, i.e., undetected by all three screening modalities at the most recent visit. In screening round 4, ultrasound had a higher AUC (0.90, 95% CI 0.74–1.00) compared to both mammography (0.58, 95% CI 0.42–0.74, *p* = 0.0026) and clinical breast examination (0.64, 95% CI 0.43–0.84, *p* = 0.0189), respectively. This pattern was similar in screening round 5, although the difference in AUCs for ultrasound (0.82, 95% CI 0.49–1.00) and mammography (0.66, 95% CI 0.34–0.99) was no longer statistically significant. The AUC for clinical breast examination (0.47, 95% CI 0.45–0.48) remained significantly lower than that of ultrasound (*p* = 0.0346) (Fig. 2).

### Risk assessment according to BOADICEA

If the BOADICEA risk model had been used with the same cut off value of ≥ 17% as used in the present study 217 (34%) of the women, of which five were diagnosed with breast cancer, would not have been eligible in this study. However, calculating the life time breast cancer risk for the five women diagnosed with breast cancer who did not reach 17% according to BOADICEA version 2, the BOADICEA version 3 captured all but one of these women with breast cancer.

## Discussion

This is the first Swedish report of yearly surveillance in a large cohort of families with breast and/or ovarian cancer. In our cohort of 632 women, a total of 30 breast cancers were detected with 10 of these detected in between screening rounds. Five of these patients were undetected gene carriers when the study started. Among the three screening modalities, mammography showed a low sensitivity and high specificity. Ultrasound showed higher sensitivity in three out of five screening rounds. The specificity of ultrasound screening improved over subsequent screening rounds. One explanation of this could be that despite the long experience of ultrasound imaging among the radiologists, the blinded procedure without having the mammograms available was novel for them, but after a few rounds their experience increased. However, only screening round 4 showed a significant difference in area under the curve between mammography and ultrasound imaging modalities. In addition, no consistent patterns in the receiver operating characteristics curves of the different screening modalities were observed across screening rounds. A possible limitation, of this study, was the low number of breast cancer cases. This low number prevented the use of elaborate statistical regression models to fully incorporate the longitudinal structure of the data (i.e., generalized mixed models) or subgroup-specific analyses stratified based on risk factors such as age or breast density. Therefore, we were not able to investigate the influence of breast density in the sensitivity analyses, although the correlation is well known [8, 14, 23]. Nevertheless, our results, in terms of sensitivity and specificity of mammography and ultrasound imaging, are similar to those reported by Kuhl et al. [11]. However, Kuhl et al. also included magnetic resonance imaging in their study and demonstrated a much higher sensitivity for magnetic resonance imaging than for mammography and ultrasound. Unfortunately, access to magnetic resonance imaging screening only became available for *BRCA1* or *BRCA2* carriers and women with a medical history of breast cancer half way through the study. Consequently, magnetic resonance imaging findings are not included in our sensitivity and specificity analyses.

The sensitivity and specificity of clinical breast examination were low in this study which is in accordance with the meta-analysis by Koster and Gotzsche [9]. Indeed, after the study was completed in 2012, clinical breast examination screening was consequently excluded in all types of surveillance program for women with increased hereditary risk of breast cancer in the Stockholm region (2 million inhabitants). In gene carriers, a yearly physician visit is optional for discussions about prophylactic surgery, psychological issues and clinical examination if desired by the patient. The positive consequences from this modification of the screening programs are of major importance since patients from all risk groups can avoid one check-up visit per year, and thus costs and time commitments. Medical providers could also benefit by avoiding these costs linked to out-patient visits. In addition, due to the high number of interval cancers (33%) and tumors with lymph node metastases (36%), and with 60% of the interval cancers detected in gene carriers, we conclude that the overall performance was poor. Therefore, it appears that the surveillance program for breast cancer mutation carriers should be modified to include screening with alternating magnetic resonance imaging and mammography and ultrasound at 6-month intervals. In 2013, updated international guidelines recommending annual magnetic resonance imaging for high risk individuals and mutation carriers [15] were also published.

The decreasing number of referrals for cytology over time presented in Fig. 2 is explained by the novel working process for the ultrasound radiologists and the clinicians in that they were obliged to consider and code all breast lesions without support from any of the other imaging modalities. However, these different numbers over time was not a limitation for the sensitivity and specificity analyses though all code 2 or less were not included in these analyses. In contrast, the mammography radiologists had long experience of reading mammograms in a standalone procedure and hence, no difference was seen throughout the study in their referrals for cytology.

Many different methods of risk estimation [2] and risk categorization have been used in screening surveillance studies [10, 11, 17]. However, categorizing women into different groups relating to their hereditary risk of breast cancer is challenging. The risk estimation in this study was based on family history pedigree patterns. This distribution of the risk groups is similar to the distribution of risk groups in a study by Kriege et al. [10] who also used the modified Claus tables [6] for risk estimation. The risk estimation model Breast and Ovarian Analysis Disease Incidence and Carrier Estimation Algorithm [BOADICEA] has replaced the Claus tables in clinical practice, but use of the latter instead of the Claus tables for risk estimation would not have affected our findings significantly (all but one woman with breast cancer would have been included in our study if the BOADICEA risk model had been used).

Our findings indicate that a modified surveillance program may be used for individuals with increased hereditary risk for breast cancer. As neither mammography nor ultrasound performed yearly were sensitive enough to detect breast cancer consistently as a standalone modality in our study, we advise women with BOADICEA lifetime risk < 20% to adhere to the population screening program with biennial mammography. For women with ≥ 20 to 29% lifetime risk according to BOADICEA a surveillance program, including annual mammography combined with ultrasound if indicated, may be sufficient. In high risk women with ≥ 30% lifetime risk in combination with high dense breasts, the same protocol as used in mutation carriers may be considered, namely screening every sixth months with magnetic resonance imaging at month 1 and mammography and ultrasound at month 6. Importantly, clinical breast examination may be omitted in all surveillance program for woman with hereditary increased risk of breast cancer, but could be optional for gene carriers.

## Electronic supplementary material

Below is the link to the electronic supplementary material.

Supplementary material 1 (XLS 29 kb)
